# Effect of Future Climate Change on Stratosphere-to-Troposphere-Exchange Driven Ozone in the Northern Hemisphere

**DOI:** 10.4209/aaqr.220414

**Published:** 2023-12

**Authors:** Shovan Kumar Sahu, Lei Chen, Song Liu, Jia Xing, Rohit Mathur

**Affiliations:** 1 Centre for Climate Research Singapore, Meteorological Service Singapore, Singapore 537054, Singapore; 2 State Key Joint Laboratory of Environmental Simulation and Pollution Control, School of Environment, Tsinghua University, Beijing 100084, China; 3 State Environmental Protection Key Laboratory of Sources and Control of Air Pollution Complex, Beijing 100084, China; 4 The U.S. Environmental Protection Agency, Research Triangle Park, NC 27711, USA; 5 Economy and Information Technology Department of Zhejiang, Zhejiang, China

**Keywords:** STE, O_3_, Northern hemisphere, Future scenarios, RCP4.5, RCP8.5

## Abstract

Future estimates of atmospheric pollutant concentrations serve as critical information for policy makers to formulate current policy indicators to achieve future targets. Tropospheric burden of O_3_ is modulated not only by anthropogenic and natural precursor emissions, but also by the downward transport of O_3_ associated with stratosphere to troposphere exchange (STE). Hence changes in the estimates of STE and its contributions are key to understand the nature and intensity of future ground level O_3_ concentrations. The difference in simulated O_3_ mixing ratios with and without the O_3_-Potential Vorticity (PV) parameterization scheme is used to represent the model estimated influence of STE on tropospheric O_3_ distributions. Though STE contributions remain constant in Northern hemisphere as a whole, regional differences exist with Europe (EUR) registering increased STE contribution in both spring and winter while Eastern China (ECH) reporting increased contribution in spring in 2050 (RCP8.5) as compared to 2015. Importance of climate change can be deduced from the fact that ECH and EUR recorded increased STE contribution to O_3_ in RCP8.5 compared to RCP4.5. Comparison of STE and non-STE meteorological process contributions to O_3_ due to climate change revealed that contributions of non-STE processes were highest in summer while STE contributions were highest in winter. EUR reported highest STE contribution while ECH reported highest non-STE contribution. None of the 3 regions show consistent low STE contribution due to future climate change (< 50%) in all seasons indicating the significance of STE to ground level O_3_.

## 1 INTRODUCTION

The global tropospheric ozone (O_3_) burden has increased since pre-industrial era (1860) by almost 50% (10.81 DU, or 118.0 Tg) till the year 2000 (Horowitz, 2006). Recent studies have indicated high tropospheric O_3_ levels across the world, with average mixing ratio of 50 ppb (12–67 ppb) wherein South Asian countries clocked the steepest rise with modest decline in O_3_ concentrations recorded in high income countries between 2015–2019 (https://www.stateofglobalair.org/air/O3). Exposure to high O_3_ levels may result in diseases such as dyspnea, upper airway irritation, coughing, and chest tightness (Carlisle and Sharp, 2001). As per global burden of disease study (GBD, 2019), ambient O_3_ pollution resulted in 36,5000 (175,000–564,000) additional mortality in 2019 (Murray et al., 2020). About 70% of these additional mortalities occurred in India and China indicating large population being exposed to very high O_3_ levels. Photochemistry involving NO_x_ and VOC precursor species and downward transport of O_3_ rich air from stratosphere are primary sources of tropospheric O_3_, while surface deposition and chemical loss and rainout constitute the primary sinks (Griffiths et al., 2020; Levy et al., 1980; Roelofs and Lelieveld, 1997; Stohl et al., 2000).

Stratosphere and troposphere are two adjoining regions in the vertical atmospheric space separated by tropopause while troposphere is the lowest layer of the atmosphere which nominally extends up to 10 km above sea level, stratosphere extends from the top of troposphere to about 50 km from the ground surface. The transport of trace chemical species between the two regions is of special interest since it can affect species concentrations in the respective layers. Stratosphere-troposphere exchange (STE) is associated with jet streams (Newell, 1963) and troposphere folds (Danielsen, 1968) and is usually driven by overturning of troposphere (Brewer, 1949; Holton et al., 1995) and mid-latitude deep convection (Fischer et al., 2003; Gray, 2003; Hegglin et al., 2004; Poulida et al., 1996). The STE results in redistribution of atmospheric O_3_, specifically, decrease in lower stratosphere and increase in upper troposphere (Hartmann et al., 2000). STE induced O_3_ contributes significantly to tropospheric column (Lelieveld and Dentener, 2000) as well as mid-tropospheric O_3_ values. Terao et al. (2008) estimated that during Spring at 500 hPa, stratospheric O_3_ can contribute up to ~40% of the O_3_ at high latitudes and ~30% in mid latitudes. Tarasick et al. (2019) in a 10-day campaign between 2005 and 2007 in Ontario and Quebec found that STE over the campaign period contributed to 3.1% O_3_ in boundary layer, 13% in lower troposphere and 34% in the middle and upper troposphere. Yang et al. (2016) observed that STE induced O_3_ is found in the extratropic with maximum STE in Northern Hemisphere occurring mostly in late spring and early summer while little seasonality was observed in southern hemisphere. Hegglin and Shepherd (2009) reported an increase of about 23% in global STE flux between 1965 and 2095 in moderate emission scenario (RCP4.5/RCP6.0) under Intergovernmental Panel on Climate Change (IPCC). Lefohn et al. (2012) quantified the contribution of STE to surface O_3_ concentration in USA and observed that STE frequently contributed to enhance surface hourly O_3_ especially at the high-elevation sites. Collins et al. (2003) estimated an overall 37% increase in STE between present (1990–1994) and future years (2090–2094).

**Fig. 1.**
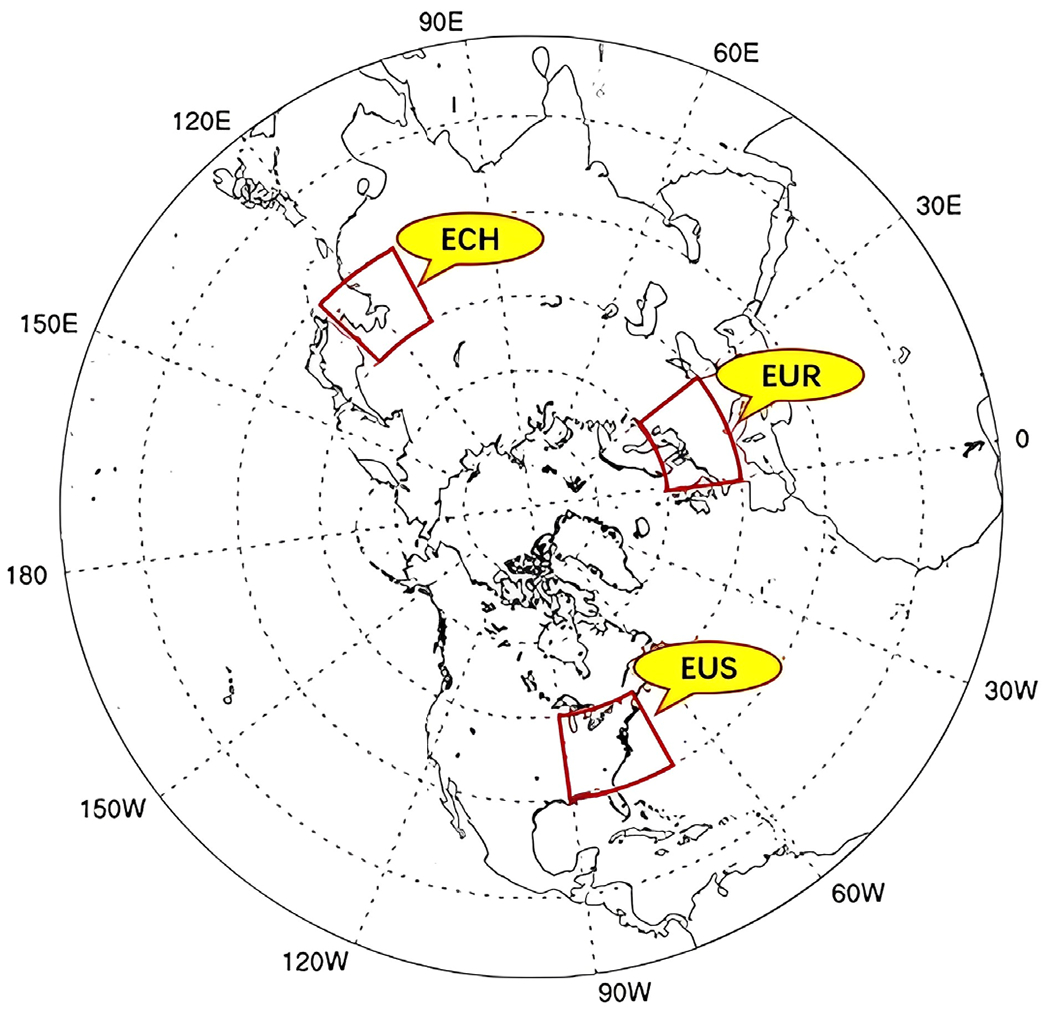
Study domain and regions: EUS, EUR and ECH.

Though anthropogenic emissions can be controlled, the non-linear O_3_ chemistry leading to its production in the atmosphere and the variability in precursor emissions from natural sources and from STE complicate the estimation and regulation for future O_3_ concentrations. As control measures reduce anthropogenic precursor emissions, there is a greater need to accurately quantify the contributions of natural precursor emissions as well as STE contributions to ground level O_3_. Changes in atmospheric dynamics under changing climate are likely to impact both the frequency and intensity of STE and consequently tropospheric O_3_ levels (Collins et al., 2003; Meul et al., 2018; Sudo et al., 2003). To understand the impacts of climate induced STE changes on O_3_ distributions across the troposphere, in this study we conduct model simulations to examine such changes associated with Representative Concentration Pathways (RCP’s) used in current climate assessments. In particular, we study the changes, characteristic and contribution of STE and non-STE meteorological processes to ground level O_3_ across the entire Northern Hemisphere with special emphasis on the three populated regions, namely Europe, Eastern China and Eastern USA as shown in Fig. 1.

## 2 METHODS

### 2.1 Model Details

Model configurations are briefly described here as they were detailed in previous studies (Xing et al., 2015). The Community Multiscale Air Quality (CMAQ) model version 5.2 (Appel et al., 2018) with AERO6 aerosol module (Appel et al., 2013) and Carbon Bond 6 gas phase chemical mechanism (Sarwar et al., 2008; Yarwood et al., 2010) was used to simulate air quality for present (2015) and future (2048–2050) years over the entire Northern Hemisphere (Mathur et al., 2017; Wang et al., 2017; Xing et al., 2015). The model utilized a horizontal grid resolution of 108 km as depicted in Fig. 1(a) while the vertical extent between the surface and 10 hPa was resolved by 64 layers (Table S1) of variable thickness. The metrological data to CMAQ was fed using outputs from Weather Research and Forecasting (WRF) model version 3.7.1 (Skamarock et al., 2008). Input meteorological data for the two RCP scenarios were obtained from climate model outputs of the NCAR’s Community Earth System Model (CESM) (Hurrell et al., 2013) used in the IPCC’s fifth assessment report (Bruyère et al., 2015). Additional information regarding different schemes used have been provided in our previous study (Sahu *et al.*, 2020). The simulation period for current year is an average of 2013, 2014, and 2015, while an average of 2048, 2049, and 2050 are used to represent the future year scenario. For brevity, from here in 2015 refers to the 3-year average for the 2013–2015 while 2050 refers to the 3-year average for 2048–2050.

### 2.2 Emission Inventory

We used the 2015 emission inventory data from Emission Database for Global Atmospheric Research (EDGAR v4.2) (Janssens-Maenhout *et al.*, 2013; Olivier et al., 1994, 1995). The biogenic emissions from Global Emission Inventory Activity (GEIA) (Guenther et al., 1995; Price et al., 1997) is used to supplement the anthropogenic emissions. The natural and anthropogenic emissions were held constant to ensure that changes in simulated O_3_ could be attributed to changes in climate induced atmospheric dynamics including STE.

### 2.3 Stratosphere Troposphere Exchange

The transport of O_3_ between upper troposphere and lower stratosphere is specified using a potential vorticity (PV)-based parameterization (Xing et al., 2016) at 50–100 hpa pressure levels. The parameterization scheme is based on several studies in the past which observed a strong relationship between potential vorticity and STE (Beekmann et al., 1994; Folkins and Appenzeller, 1996). Although future climate change may well change the meteorological parameters including the potential vorticity, but will it change the relationship between O_3_, and potential vorticity remains to be seen. Hence the same parameterization scheme was used to estimate the STE in future climate change scenario. Two scenarios are simulated to estimate the impacts of STE on tropospheric O_3_ distributions. Scenario 1 without the O_3_-PV parameterization represents the case with no stratospheric O_3_ transported to the troposphere (hereafter denoted as sim_noSTE). Scenario 2 invokes the O_3_-PV parameterization with all aspects similar to Scenario 1 representing the influences of stratosphere-troposphere exchange on tropospheric O_3_ distributions as previously described and evaluated in Xing et al. (2016) and Mathur et al. (2017) (denoted as sim_STE_PAR). The difference in simulated O_3_ mixing ratios between Scenario 2 and Scenario 1 (sim_STE_PAR-sim_noSTE) then represent the model estimated influence of STE on tropospheric O_3_ distributions. The O_3_ concentration hence referred here after is the surface O_3_ concentration unless otherwise specified.

### 2.4 STE Contribution to O_3_

STE contribution to O_3_ is discussed in 2 ways: 1) STE contribution to O_3_ in present and future years, 2) STE contribution to O_3_ due to future climate change.

1) STE contribution to O_3_ in base and future years

The present and future year STE contribution is estimated as in expression 1:

“pv” indicates simulation with O_3_-PV parameterization turned on while “nopv” indicates simulation without it

(1) $$\frac{{\text{O}}_{3}2015pv-{\text{O}}_{3}2015nopv}{{\text{O}}_{3}2015pv};\frac{{\text{O}}_{3}2050pv-{\text{O}}_{3}2050nopv}{{\text{O}}_{3}2050pv}$$

where ${\text{O}}_{3}2015\text{pv}$ and ${\text{O}}_{3}2050\text{pv}$ is defined as O_3_ concentration in present (2015) and future scenario (2050), respectively.

2) STE contribution to O_3_ due to future climate change

In order to estimate STE contribution to O_3_ due to future climate change, we first estimate the contribution of non-STE meteorological processes to O_3_ due to climate change. Since while simulating, only meteorology between 2050 and 2015 changes and everything else remain constant, the contribution of meteorology to O_3_ due to climate change can be estimated as in expression 2:

(2) $$\frac{{\text{O}}_{3}2050nopv-{\text{O}}_{3}2015nopv}{{\text{O}}_{3}2050pv-{\text{O}}_{3}2015pv}$$

where ${\text{O}}_{3}2050\text{pv}-{\text{O}}_{3}2015\text{pv}$ is defined as O_3_ concentration due to climate change The STE contribution to O_3_ due to climate change is then estimated as in expression 3:

(3) $$100-\%\text{Meteolorology contribution to}\;{\text{O}}_{3}\;\text{due to climate change}$$

The significance of the change in STE contribution in future years due to climate change or the difference in STE contributions in different scenarios (RCP4.5 and RCP8.5) is estimated using Student’s t-test (Student, 1908). The null hypothesis assumes that sample means in case of future and current year or in different scenarios (RCP4.5 and RCP8.5) are from the same population and is rejected when the critical significance value (*p*) is less than 0.05. Thus, rejection of null hypothesis reaffirms that the difference between the sample means is significant. However, it should be noted that the significance of STE and non-STE parameters is studied separately here to understand how these parameters individually impact surface O_3_.

## 3 RESULTS AND DISCUSSION

### 3.1 Model Performance

The model outputs were compared with observations across the Northern Hemisphere in order to elucidate the performance of the model. Ground-level O_3_ observational data in China were obtained from CN-AQI (China Air Quality Index, http://106.37.208.233:20035/) while for Europe the observational data was from EU-EMEP (European Monitoring and Evaluation Programme, http://www.emep.int) and EU-AIRBASE (The European air quality database, http://acm.eionet.europa.eu/databases/airbase/). The CN-AQ and EU-AIRBASE monitoring stations are mostly located in urban centers while EU-EMEP is mostly located in suburbs. Additionally, vertical profile of O_3_ as well as the surface observations in remaining parts of the world were obtained from WOUDC (The World O_3_ and Ultraviolet Radiation Data Centre, http://www.woudc.org/). The model performance metrics were estimated based on hourly data averaged across grids covering monitoring stations with the observed data being obtained from various sources, in other cases data is averaged over all the grids cells in a region. The mean bias (MB) in Table 1. refers to a minor overall over-prediction at WODUC sites (4.49 ppb) located across the world as well as in urban centers of China (9.33 ppb) and Europe (5 ppb) while a minor under prediction is observed in the European suburbs (−3.62 ppb). The normalized mean bias (NMB) doesn’t meet the criteria for O_3_ (< ±15%) in urban centers of China (39.8%) and Europe (18.2%) while WODUC (13.3%) and European suburban (−9.1%) sites meet the criteria limits as suggested by Emery et al. (2017).

The NMB values were mostly similar as reported in our previous study Sahu *et al.* (2020). It should be noted that since these criteria limits were derived based on modeling studies utilizing much finer grid sizes, these threshold values should be viewed as guidelines rather than definitive performance limits (Zhang et al., 2014). In order to further validate the O_3_-PV parameterization, we compared the simulated vertical O_3_ distribution using the O_3_-PV parameterization scheme with simulated concentration without parameterization scheme as well as observed WOUDC vertical O_3_ measurements (Fig. S1). The vertical O_3_ distribution obtained using the O_3_-PV parameterization scheme (red line) is close to observed O_3_ measurements (black line) while the simulation without the O_3_-PV scheme (blue line) grossly underestimates the observed values. As indicated previously (Xing et al., 2016; Mathur et al., 2017; Hogrefe et al., 2018) and by these summary-analysis for 2015, the model reasonably well captures both the vertical and horizontal variations in observed tropospheric O_3_.

### 3.2 STE Contribution to Surface O_3_ Concentration in Base Year

**Fig. 2.**
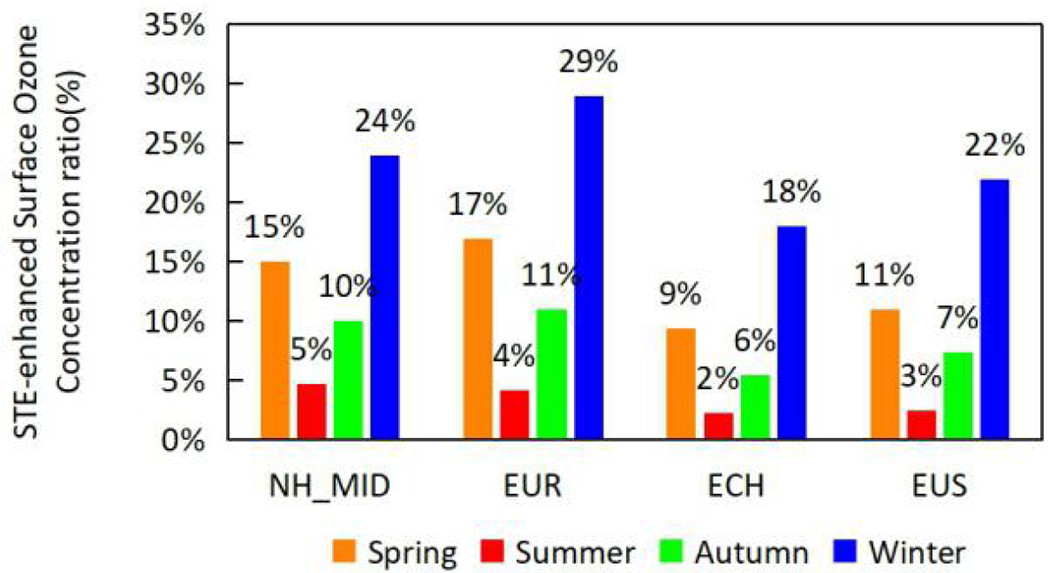
STE contribution to ground level O_3_ (%) in regions across Northern Hemisphere. *NH_MID refers to mid latitudes (30°N–60°N) in Northern Hemisphere.

STE contribution (expression 1) to surface O_3_ (O_3_2015pv) was noted to be higher in winter (Dec, Jan, Feb) and spring (Mar, Apr, May) especially in the mid latitudes (30°N–60°N) of Northern Hemisphere (NH_MID) as compared to autumn (Sep, Oct, Nov) and summer (Jun, Jul, Aug). The average contribution of STE to surface O_3_ in NH_MID was estimated to be 24% and 15%, respectively, in winter and spring (Fig. 2 and Fig. S2(a)).

Similar trend was consistent across the regions with Europe (EUR), Eastern China (ECH) and Eastern USA (EUS) showing high STE contributions in winter (29%, 18%, and 22%) and spring (17%, 9%, and 11%). This corresponds well to the estimates in our earlier analysis (Xing et al., 2016), in which the estimated STE contributions were 32.3%, 15.4% and 25.5% in winter and 21%, 10.5%, and 17% in spring, for the EUR, ECH and entire Continental U.S. The absolute STE concentration as well as its contribution to surface ozone is higher (Fig. 2 and Fig. S2(a)) mainly due to deeper intrusion of O_3_ from upper layers especially in latitudes higher than 20°N as shown in the vertical distributions in supplementary information (Fig. S2(b)). Likewise, higher STE contributions to surface O_3_ in EUR can be directly inferred from the deeper intrusion of O_3_ from upper layers in EUR region as compared to that in ECH or EUS (Fig. S3). Further, higher hourly O_3_ concentration and Planetary Boundary Layer (PBL) height in EUR as compared to ECH or EUS in spring and winter seasons explains the higher O_3_ entrainment from upper layers to the surface which results in an increased STE induced surface O_3_ in EUR.

Notably, the hourly O_3_ concentration and PBL have similar trends in both winter and spring seasons in all regions with maximum value in afternoon and lower values in morning and evening. An analysis of STE contribution to O_3_ concentration ranges (low (0–20.41 ppb), moderate (20.41–40.82 ppb), moderately high (40.82–61.23 ppb) and high (61.23–81.63 ppb)) suggested absolute STE contributions to O_3_ is less during high O_3_ days (61.23–81.64 ppb) as compared to that in low O_3_ days (< 61.23 ppb), especially in ECH (Table S2). The STE contribution during high O_3_ period in EUS and EUR however couldn’t be verified since the maximum O_3_ concentration range is below 40.82 ppb in both regions. However, a similar analysis for the entire mid latitude region (30°N–60°N) revealed that the trend in the entire region was similar to that of ECH (Table S3). Further, evaluation of STE contribution to O_3_ on good (Level 1: Air Pollution Index 0–50) and bad (Level 5: Air Pollution Index > 300) air quality days in ECH revealed that STE contributed O_3_ on good air quality days was higher (5.8–6.8 ppb) as compared to that in bad air quality days (1.8–5.3 ppb) which can be attributed to higher PBL height on good air quality days resulting in higher entrainment of O_3_ of stratospheric origin from upper layers (Fig. S4). It should be noted however that good and bad air quality index is based on the maximum index value among the criteria pollutants. Since particulate matter is usually the dominating index, hence ozone concentration on a good air quality day might not be necessarily low. Higher STE driven O_3_ at noon especially when in-situ chemistry derived O_3_ is high, will further add on its levels. However, on bad air quality days STE induced O_3_ is low and hence a smaller contributor to air quality non-attainment, but nevertheless a modulator of secondary pollutant formation (Chen et al., 2020).

### 3.3 STE Contribution to Surface O_3_ Concentration in Future Year Scenario

**Fig. 3.**
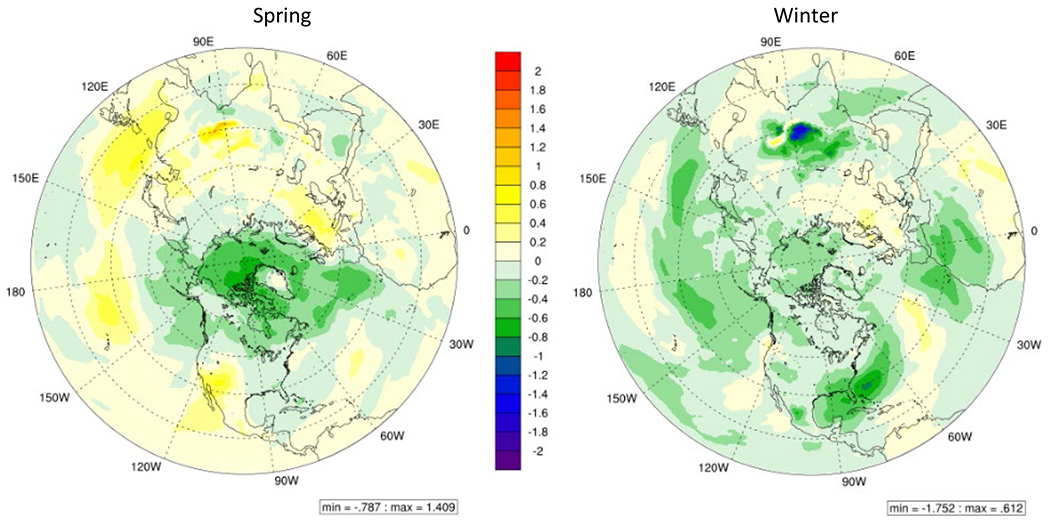
Change in STE contribution to surface O_3_ concentration (ppb) in 2050 as compared to that in 2015 in RCP8.5 scenario (2050–2015).

O_3_ concentrations in 2050 were simulated using meteorology from 2 different climate scenarios, RCP8.5 and RCP4.5 with and without O_3_-PV parameterization. In RCP8.5 scenario the overall STE contributions (expression 1) to O_3_ (O_3_2050pv) across the entire Northern Hemisphere remains the same as in 2015 during spring (6.8% (*p* < 0.05)) while it reduces marginally by 0.7% (*p* < 0.05) in 2050 as compared to that in 2015 in winter (Fig. 3).

EUR is the only region to witness growth in STE contributions in both spring and winter by 1% (*p* < 0.05) and 1.1% (*p* < 0.05) respectively. ECH registered an increase in STE contribution in spring by 0.5% (*p* < 0.05) while it reduced by 0.6% (*p* < 0.05) in winter, EUS recorded decrease in both spring and winter by 0.3% (*p* < 0.05) and 0.9% (*p* < 0.05) respectively. The change in STE is influenced by a combination of several factors including temperature, tropopause height, geopotential height, polar front jet, and hence the influence of the same on STE isn’t linear. We cannot comment on the dominating factor in a region since it would require a series of sensitivity analysis which is outside the scope of the present study. Hence, we limit ourselves to discussing the combined effect of the above-mentioned factors. In the following lines we first discuss change in the above-mentioned meteorological parameters due to future climate change followed by the corresponding impact of those parameters on STE.

**Fig. 4.**
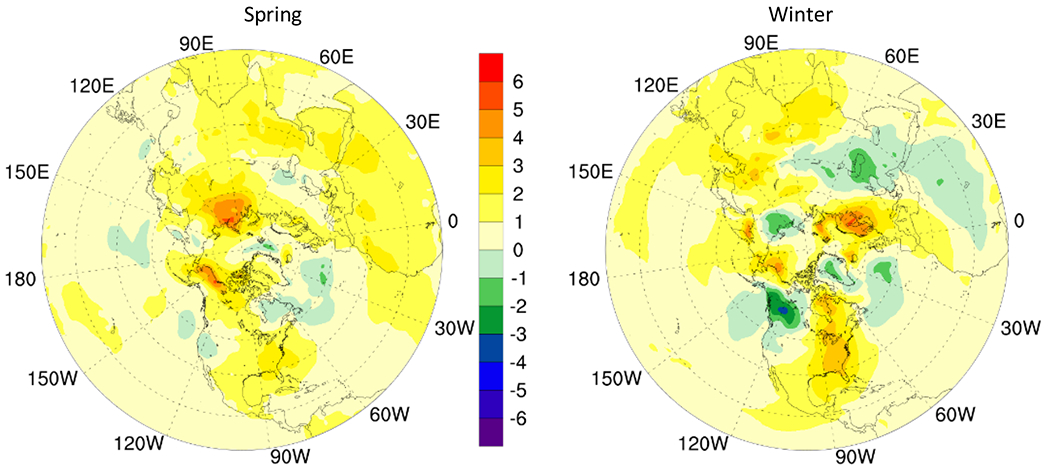
Change in surface temperature (K) in 2050 RCP8.5 scenario as compared to in 2015.

The increase in temperature in 2050 under RCP8.5 scenario is obvious in all regions barring high latitude areas near the arctic circle resulting in increased STE induced O_3_ due to stronger vertical convection because of increased temperature gradient (Fig. 4).

**Fig. 5.**
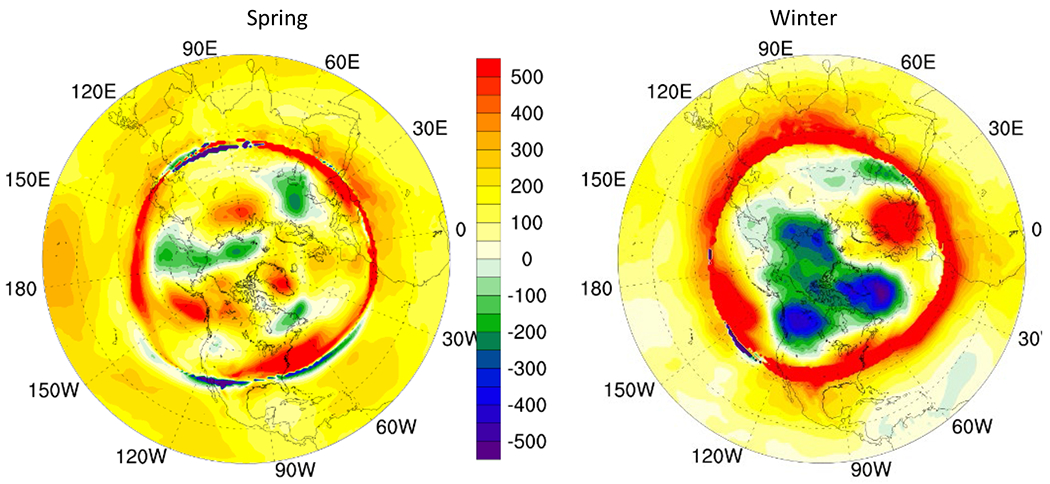
Change in tropopause height (m) in 2050 RCP8.5 scenario as compared to in 2015.

Additionally, increase in tropopause height in mid-latitudes especially in spring can result in reduced O_3_ concentration in upper troposphere in 2050 as compared to that in 2015 (Fig. 5).

**Fig. 6.**
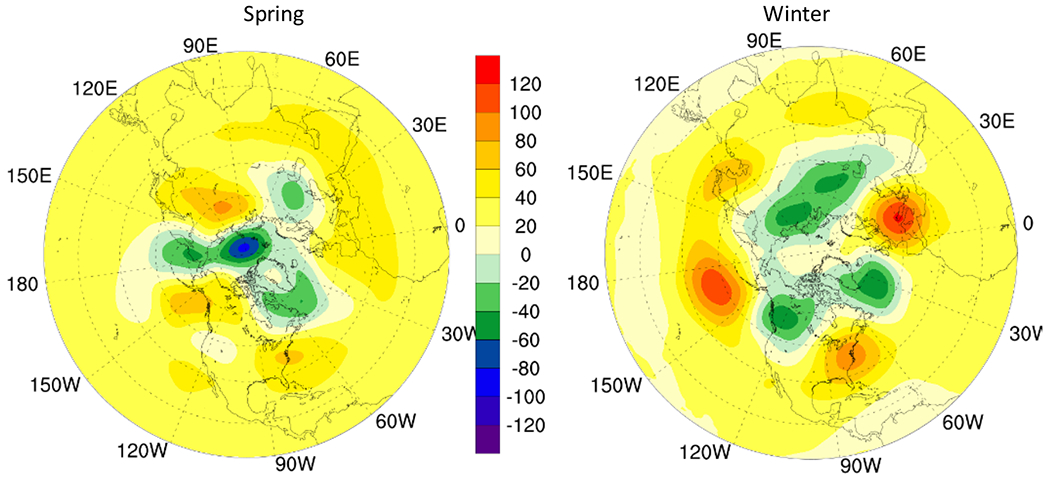
Change in 500 hPa geopotential height in 2050 RCP8.5 scenario as compared to in 2015. (gpm)

The change in tropopause height in polar regions is more apparent with increase in spring in most areas and an overall reduction in winter indicating reduced O_3_ concentration in spring and an increased O_3_ concentration in upper troposphere in winter. The 500 hPa geopotential height increases in mid-latitude during winter and spring resulting in enhanced STE induced O_3_ while weakened geopotential height in the arctic circle inhibits downward transport of O_3_ (Fig. 6).

**Fig. 7.**
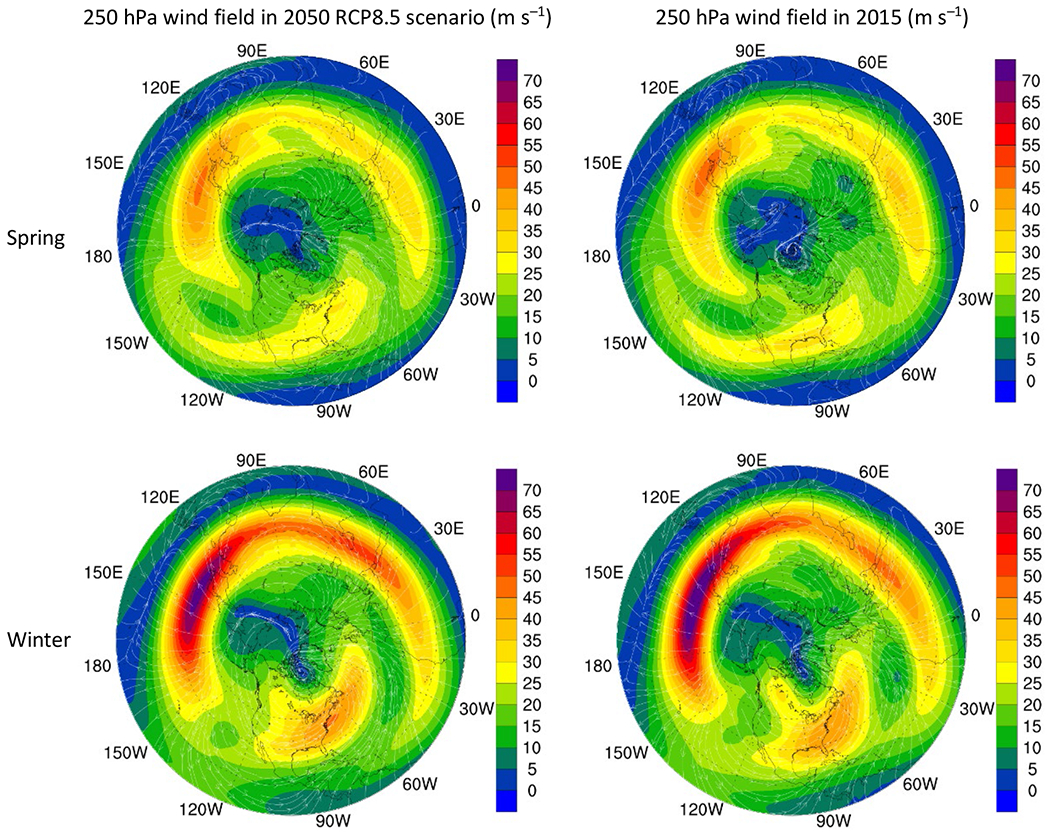
Wind field in 2050 RCP8.5 scenario as compared to in 2015.

The mixing of polar front jet and subtropical jet stream results in O_3_ transport from stratosphere to troposphere in middle latitudes (Price and Vaughan, 1993; Ren et al., 2011; Van Haver et al., 1996). Thus, the 250 hPa wind field (jet stream) intensity and extent also have a strong influence on transport of O_3_ (Allam and Tuck, 1984; Langford, 1999; Škerlak et al., 2014; Zachariasse et al., 2000) which weakened in 2050 in most regions as compared to that in 2015 (Fig. 7).

Some of the meteorological factors discussed above aid to downward transport of O_3_ while others result in reduction of downward transport. The nature of these factors may differ regionally as well as may change in future years; thus, the combined effect of both factors (favorable and non-favorable affecting downward transport of O_3_) may result in the cancellation of their effects when entire Northern Hemisphere is considered. This may explain the almost similar STE contributions to O_3_ in 2015 and 2050 in spring apart from a very modest reduction in winter. This is because when the entire Northern Hemisphere is taken into consideration, the increase in downward transport in one region might be counter-balanced by decrease in another region. The EUR region was the only region to exhibit a growth in STE induced O_3_ in both seasons which can be explained from the fact that apart from increased tropopause height, meteorological factors such as geopotential height at 500 hPa, temperature and wind field intensity at 250 hPa were conducive for downward transport of O_3_ in both seasons.

**Fig. 8.**
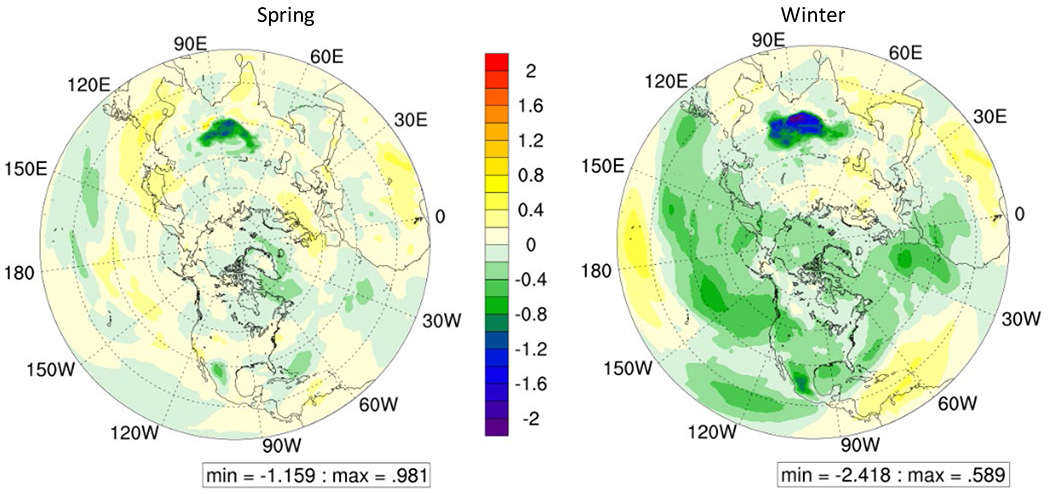
Change in STE contribution to surface O_3_ concentration in 2050 between RCP8.5 and RCP4.5 scenario (RCP8.5–RCP4.5).

Comparison of hourly STE induced O_3_ in 2015 and 2050 (Fig. S9) reveals that the hourly trend remains similar with highest STE induced O_3_ in afternoon and lowest at night which follows similar trend of PBL height. Higher PBL height results in higher transport of O_3_ to lower layers. STE induced O_3_ in 2050 was higher compared to 2015 during both winter and spring in EUR, while in ECH, the STE induced O_3_ in 2050 was higher than that in 2015 only in spring. Similar magnitude of STE induced O_3_ in 2015 and 2050 was observed in ECH in winter and in EUS in spring while during winter in EUS, STE induced O_3_ in 2050 was lower than in 2015. In order to further understand the uncertainties in estimation of STE induced O_3_ in future climate scenario, differences in STE between RCP8.5 and RCP4.5 in the year 2050 (Fig. 8) were investigated.

Compared to the RCP4.5 scenario, STE induced O_3_ is lower in RCP8.5 scenario in winter, while in spring overall STE induced O_3_ on land masses is higher in RCP8.5 scenario. Seasonal-mean STE induced O_3_ was noted to increase in the ECH by 0.1 ppb (*p* < 0.05) and in EUR region by 0.2 ppb (*p* < 0.05) in spring while in winter it increased by 0.1 ppb (*p* < 0.05) in both the regions in RCP8.5 scenario. The STE increase in spring and winter in both regions can be explained from the favorable meteorological conditions, i.e., increased temperature (Fig. S5), reduced tropopause height (Fig. S6), increased geopotential height (Fig. S7) and increased or similar jet stream intensity at 250 hPa (Fig. S8) in the region except that in case of tropopause height it increases in RCP8.5 scenario when compared to RCP4.5 scenario in winter. EUS recorded reduction of STE induced O_3_ by 0.2 ppb (*p* < 0.05) in winter while STE induced O_3_ increased in spring by 0.1 ppb (*p* < 0.05) in RCP8.5 scenario as compared to that in RCP4.5 scenario. This can again be explained based on the meteorological conditions with the only difference between the two seasons being in tropopause height which reduces in winter which might have resulted in reduced STE induced O_3_ in RCP4.5 scenario. The analysis of future climate scenarios suggests that although the STE induced O_3_ across the Northern Hemisphere on average may not change much, levels of O_3_ with stratospheric origin could increase in the mid latitude regions comprising of EUR, ECH, and EUS which also are regions with high population density, thereby potentially resulting in increased population exposure to O_3_ under these future climate scenarios. Given that in-situ O_3_ production from emissions of precursor species is also expected to increase as a result of increased intensity of atmospheric chemistry from rising temperatures in these scenarios (Jacob and Winner, 2009; Nolte et al., 2008), the cumulative impacts of climate change on O_3_ pollution both from changes in atmospheric chemistry and stratosphere-troposphere exchange should be more rigorously assessed.

### 3.4 Seasonal Meteorology and STE Contribution to O_3_ Due to Future Climate Change

Since the only difference in the simulations for 2015 and 2050 was meteorology fields, a ratio of change in O_3_ concentrations between 2015 and 2050 without the O_3_-PV parameterization scheme to the one with the parameterization scheme provides an estimate of the relative contribution of meteorology to O_3_ concentration due to future climate change. STE contribution is the only other source apart from non-STE meteorological processes in these simulations and hence can be estimated by subtracting differences caused by meteorological variables from the total changes (the total changes caused by both factors should sum to 100% (expressions 2 and 3)). Table 2 lists the seasonal contribution of STE and meteorology to change in O_3_ concentration due to future climate change in the 3 regions.

Contributions in both RCP4.5 and RCP8.5 scenarios were estimated to consider the uncertainties associated with future year simulations. In fact, both scenarios suggest almost similar contributions by STE and non-STE meteorological processes to the O_3_ change and hence we report the contribution range as of range of STE contribution in RCP4.5 and RCP8.5 scenario (min(RCP4.5, RCP8.5)–max(RCP4.5, RCP8.5)). Summer had the largest O_3_ contribution from non-STE meteorological processes (EUR: 40-41%, EUS: 63–71%, ECH: 70–81%) while winter had the largest contribution from STE (EUR: 73–76%, EUS: 53–63%, ECH: 71%). High contribution from non-STE meteorological processes to change in O_3_ in summer can be attributed to higher temperatures which is conducive for photochemical reactions between the precursors. Likewise, high STE contributions in winter can be attributed to higher intrusion of O_3_ from upper layers as discussed in Section 3.2. The highest contribution from non-STE meteorological processes (50–81%) was observed in ECH in all seasons except in winter with contribution in summer being the highest (70–81%). EUR, on the other hand, experienced the highest STE contributions in all seasons (Winter: 73–76%, Spring: 53–78%, Summer: 59–60%) except in autumn. EUR was also observed to be the region which was affected the least by meteorological processes (Winter: 24–27%, Spring: 22–47%, Summer: 40–41%, Autumn: 35–41%), the same for region affected least by STE (< 50% in all seasons for any region) couldn’t be determined due to absence of any specific trend. This also point out that STE contributions are significant in all the 3 regions and hence apart from climate penalty, STE contributions must be factored in when analyzing the effects of future climate change. It should be noted that although the total STE contribution to surface O_3_ is significant i.e., *p* < 0.05, the absolute value may not be substantial. However, these values maybe subjected to uncertainties related to emission pathways. Hence there is a possibility that in future emission pathways, the absolute contributions from STE can further increase.

## 4 CONCLUSION

The major conclusions of this study can be underlined in 5 points.

1. STE contribution to surface O_3_ in winter and spring in mid latitudes is significant.
2. STE contribution to surface O_3_ is higher in low O_3_ days and vice versa.
3. Spatial variation is observed in future STE contribution to surface O_3_ in Northern Hemisphere which would remain constant as a whole except increase in EUR in spring and winter and in ECH in spring.
4. STE contribution to O_3_ is higher in RCP8.5 scenario as compared to in RCP4.5 scenario except in EUS in winter.
5. EUR experienced the highest STE contribution while ECH experienced the highest non-STE meteorological contribution to O_3_ due to climate change.

The current study depicts the importance of STE contribution to surface O_3_ concentration specifically in the mid latitudes of the Northern Hemisphere. The study also indicates the urgency in dealing with climate change indicating that business as usual scenario will result in higher STE contribution to surface O_3_ as compared to in RCP4.5 scenario in all the 3 regions (except in EUS where it reduced by 0.3 ppb only in winter). The importance of STE contributions to O_3_ due to future climate change can be further derived from the fact that none of the 3 regions reported consistent low STE contributions (< 50%) in all seasons.

However, like all studies the current paper too have its fair share of shortcomings. Future climate studies are bound to be associated with uncertainties related to emission pathways although average of 3 years of future simulation (2048–2050) was done in this study in order to average out the uncertainties. The current study also doesn’t take into consideration future anthropogenic or natural emission changes which can completely overturn the results presented in the paper. Future research is thus required which considers expected anthropogenic emission increase or cuts as well as natural emissions to better represent the effect of STE contribution to O_3_ due to climate change.

## Supplementary Material

Supplement1

## Figures and Tables

**Table 1.** Model performance across Northern Hemisphere.

		obs (ppb)	sim^a^ (ppb)	N (pairs)	MB (ppb)	NMB (%)	R
O_3_	CN-AQI	28.52	37.86	17443	9.34	39.8	0.2
	EU-AIRBASE	27.55	32.55	2047	5.00	18.2	0.3
	EU-EMEP	35.92	32.30	704	−3.62	−9.1	0.6
	WOUDC	36.94	41.48	167	4.49	13.3	0.6

a sim refers to the simulated concentration (annual average).

**Table 2.** Contribution of non-STE meteorological parameters and STE to change in O_3_ concentration due to climate change.

Regions	% contribution of non-STE Meteorology (O_3_2050nopv – O_3_2015nopv)/(O_3_2050pv – O_3_2015pv)	% contribution of STE (100 – % Met contrib.)	% contribution of non-STE Meteorology (O_3_2050nopv – O_3_2015nopv)/(O_3_2050pv – O_3_2015pv)	% contribution of STE (100 – % Met contrib.)
	
	RCP4.5		RCP8.5	
Winter				
Europe	27	73	24	76
Eastern USA	47	53	37	63
Eastern China	29	71	29	71
Spring				
Europe	47	53	22	78
Eastern USA	34	66	52	48
Eastern China	50	50	51	49
Summer				
Europe	41	59	40	60
Eastern USA	63	37	71	29
Eastern China	70	30	81	19
Autumn				
Europe	41	59	35	65
Eastern USA	29	71	27	73
Eastern China	55	45	50	50

* pv and nopv refers to simulation with and without vertical parameterization being turned on.
