# Supplementary material for: Effect of Future Climate Change on Stratosphere-to-Troposphere-Exchange Driven Ozone in the Northern Hemisphere
Source: Aerosol Air Qual Res. Author manuscript; Available in PMC 2024 Dec 1. (PMC10802885; doi:10.4209/aaqr.220414)
Supplement: Supplement1 [file NIHMS1954153-supplement-Supplement1.pdf]

# Effect of Future Climate Change on Stratosphere-to-Troposphere-Exchange Driven Ozone in the Northern Hemisphere

Shovan Kumar Sahu[1,2,3], Lei Chen[2,3], Song Liu[2,3,5], Jia Xing[2,3*], Rohit Mathur[4]

[1]Centre for Climate Research Singapore, Meteorological Service Singapore, Singapore 537054, Singapore

[2]State Key Joint Laboratory of Environmental Simulation and Pollution Control, School of Environment, Tsinghua University, Beijing 100084, China

[3]State Environmental Protection Key Laboratory of Sources and Control of Air Pollution Complex, Beijing 100084, China

[4]The U.S. Environmental Protection Agency, Research Triangle Park, NC 27711, USA

[5]Economy and Information Technology Department of Zhejiang, China

[*]Corresponding Author's Email: xingjia@tsinghua.edu.cn

Table S1. Parameter settings for the running models ($P_{top}$=1000Pa).

| Parameter | Setting |
|---|---|
| sigma (σ) | 1.000, 0.995, 0.9901, 0.9851, 0.9802, 0.9753, 0.9703, 0.965, 0.9595, 0.9537, 0.9476, 0.9412, 0.9344, 0.9272, 0.9195, 0.9113, 0.9024, 0.8929, 0.8826, 0.8716, 0.8596, 0.8467, 0.8327, 0.8177, 0.8014, 0.7839, 0.7652, 0.7451, 0.7238, 0.7011, 0.6772, 0.6521, 0.6259, 0.5988, 0.5708, 0.5421, 0.5129, 0.4835, 0.4539, 0.4244, 0.3953, 0.3665, 0.3384, 0.3111, 0.2847, 0.2594, 0.2351, 0.212, 0.19, 0.167, 0.1478, 0.1301, 0.1138, 0.0988, 0.0851, 0.0726, 0.0611, 0.0507, 0.0412, 0.0326, 0.0247, 0.0176, 0.0112, 0.0053, 0.000 |

Table S2. % (absolute concentration) STE contribution to Ozone at different $O_3$ concentration levels in the 3 regions

| Regions | 0-40 (μg/m$^3$) | 40-80 (μg/m$^3$) | 80-120 (μg/m$^3$) | 120-160 (μg/m$^3$) |
|---|---|---|---|---|
| Winter | | | | |
| Europe | | 33.62 (20.67) | | |
| Eastern USA | 34.35 (12.56) | 23.51(15) | | |
| Eastern China | 23.59 (8.56) | 21.52 (13.44) | 14.50 (13.50) | 11 (14) |
| Spring | | | | |
| Europe | | 33.71 (20.80) | | |
| Eastern USA | 34.32 (12.60) | 23.55 (15) | | |
| Eastern China | 23.72 (8.62) | 21.65 (13.56) | 14.78 (13.74) | 11 (14) |
| Summer | | | | |
| Europe | | 33.71 (20.80) | | |
| Eastern USA | 34.32 (12.6) | 23.55 (15) | | |
| Eastern China | 23.55 (8.52) | 21.75 (13.60) | 14.70 (13.64) | 11 (14) |
| Autumn | | | | |
| Europe | | 33.73 (20.82) | | |
| Eastern USA | 34.36 (12.58) | 23.48 (15) | | |
| Eastern China | 23.70 (8.61) | 21.60 (13.51) | 14.70 (13.67) | 11 (13.96) |

Table S3: % (absolute concentration) STE contribution to Ozone at different $O_3$ concentration levels in mid-high latitude region (30ºN-60ºN)

| 0-40 (μg/m$^3$) | 40-80 (μg/m$^3$) | 80-120 (μg/m$^3$) | 120-160 (μg/m$^3$) |
|---|---|---|---|
| 28.48 (9.78) | 26.65 (17.54) | 19.78 (17.19) | 8.29 (10.53) |

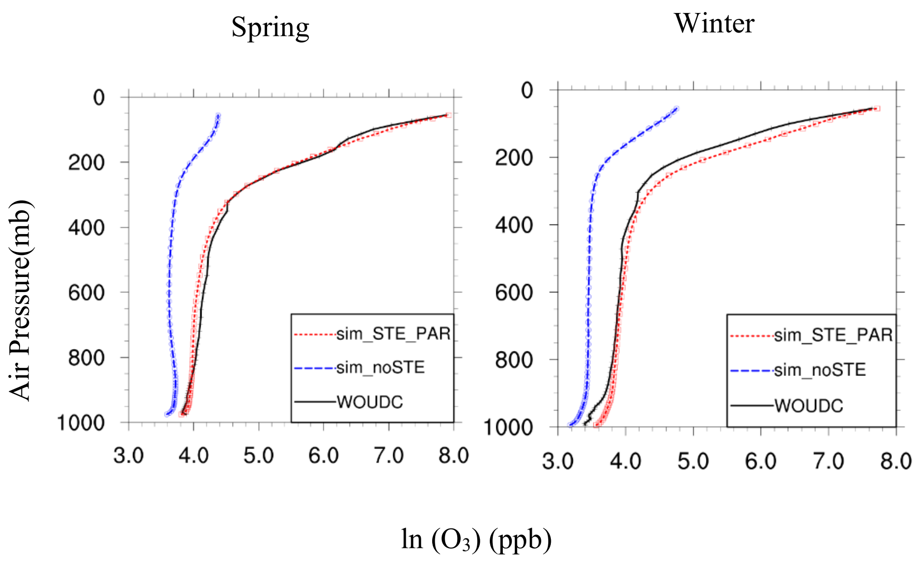


Figure S1. Seasonal variation in vertical profile of ozone concentration in northern hemisphere.

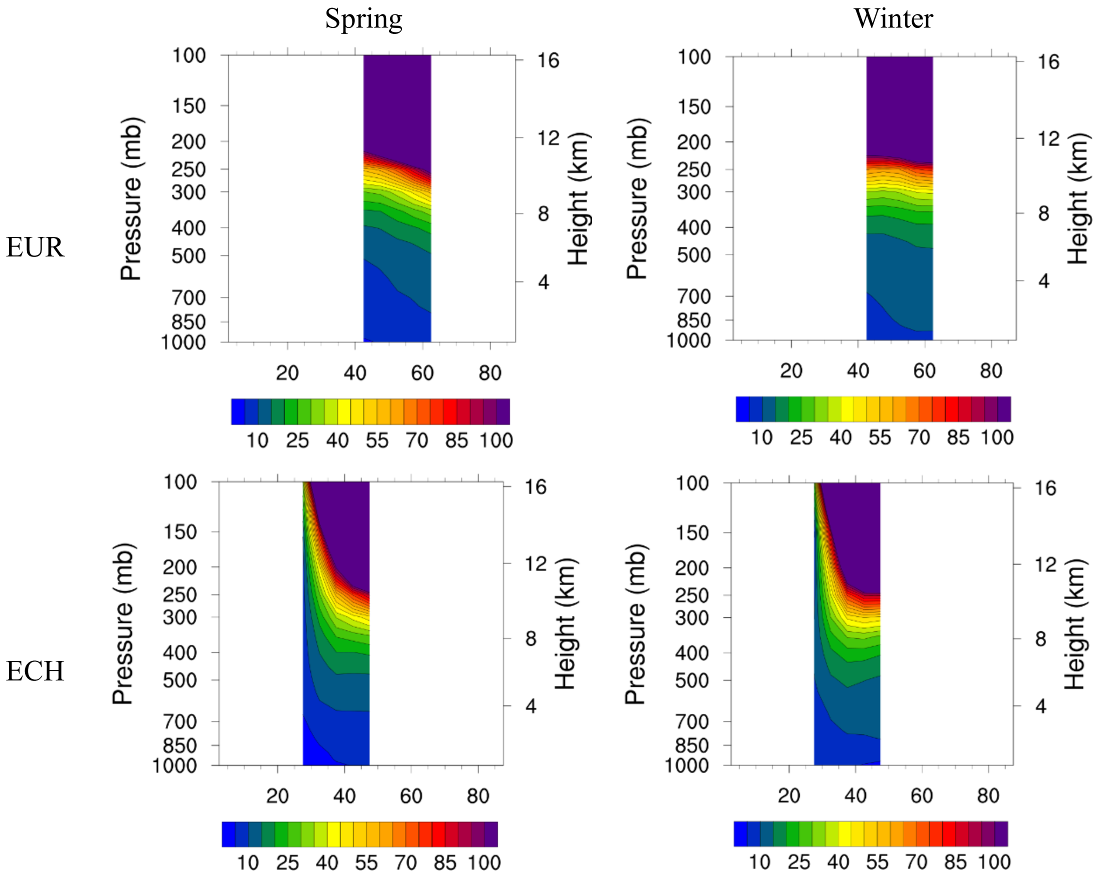

EUS

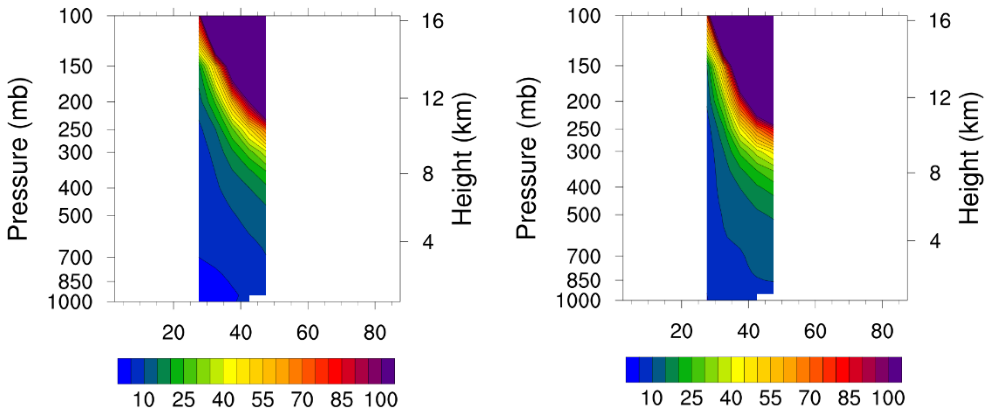


Figure S2. Zonal average ozone vertical plot for 2015 (ppb)

EUR

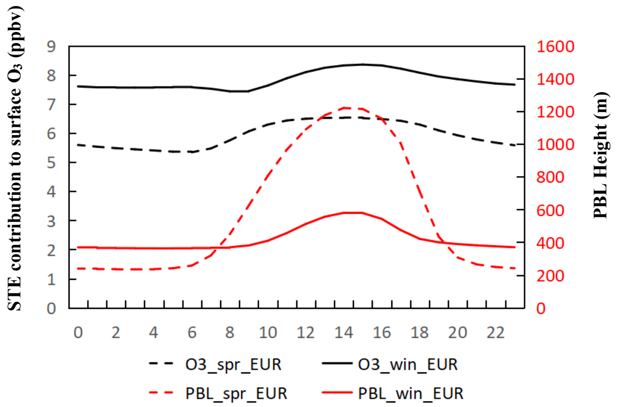


ECH

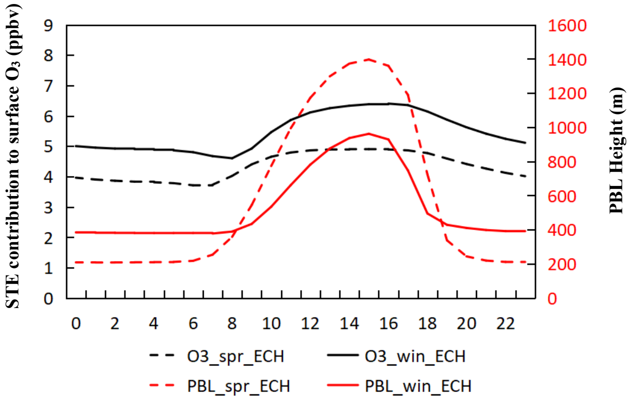

EUS

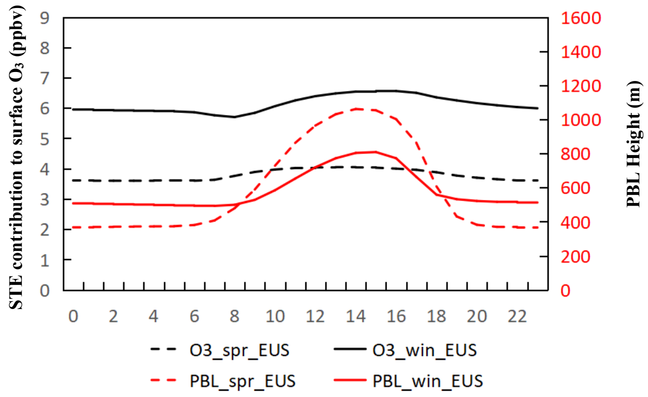


Figure S3. Hourly STE induced ozone and PBL in 2015

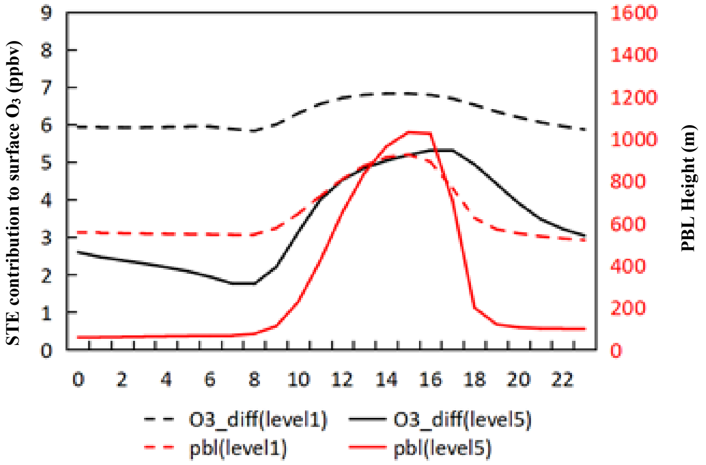


Figure S4. Hourly STE induced ozone and PBL under different air quality indexes in 2015

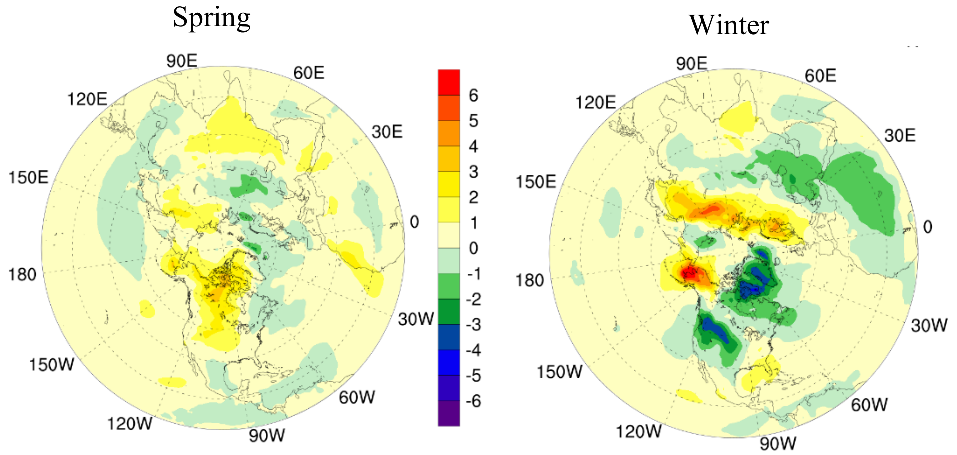


Figure S5. Change in surface temperature in 2050 RCP8.5 scenario as compared to in RCP4.5 (K)

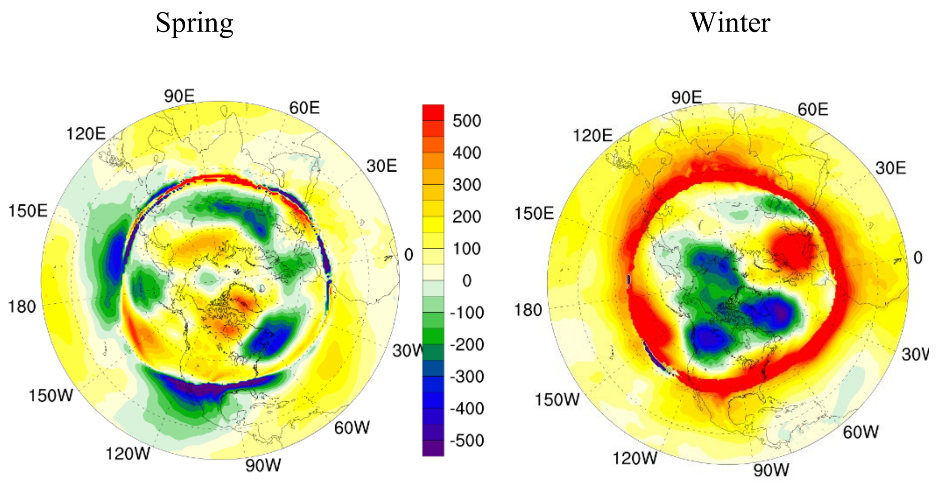


Figure S6. Change in tropopause height in 2050 RCP8.5 scenario as compared to in RCP4.5 (m)

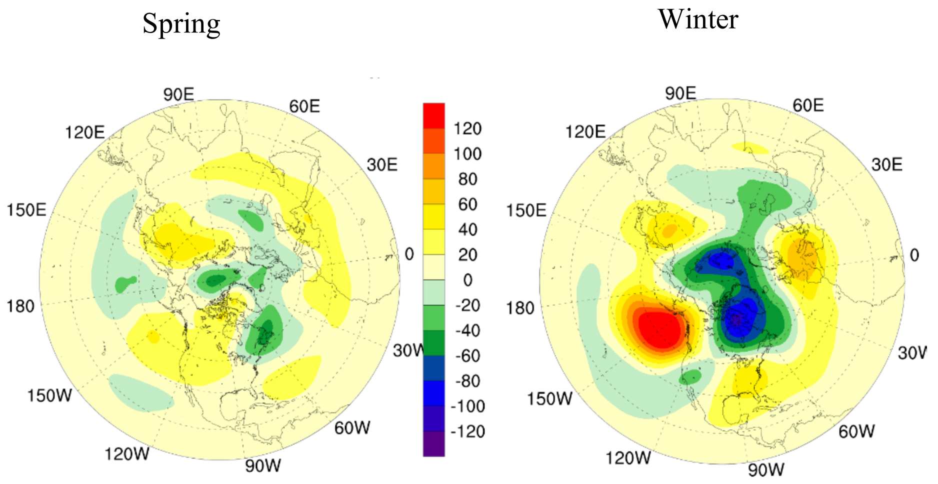


Figure S7. Change in 500hPa geopotential height in 2050 RCP8.5 scenario as compared to in RCP4.5 (gpm)

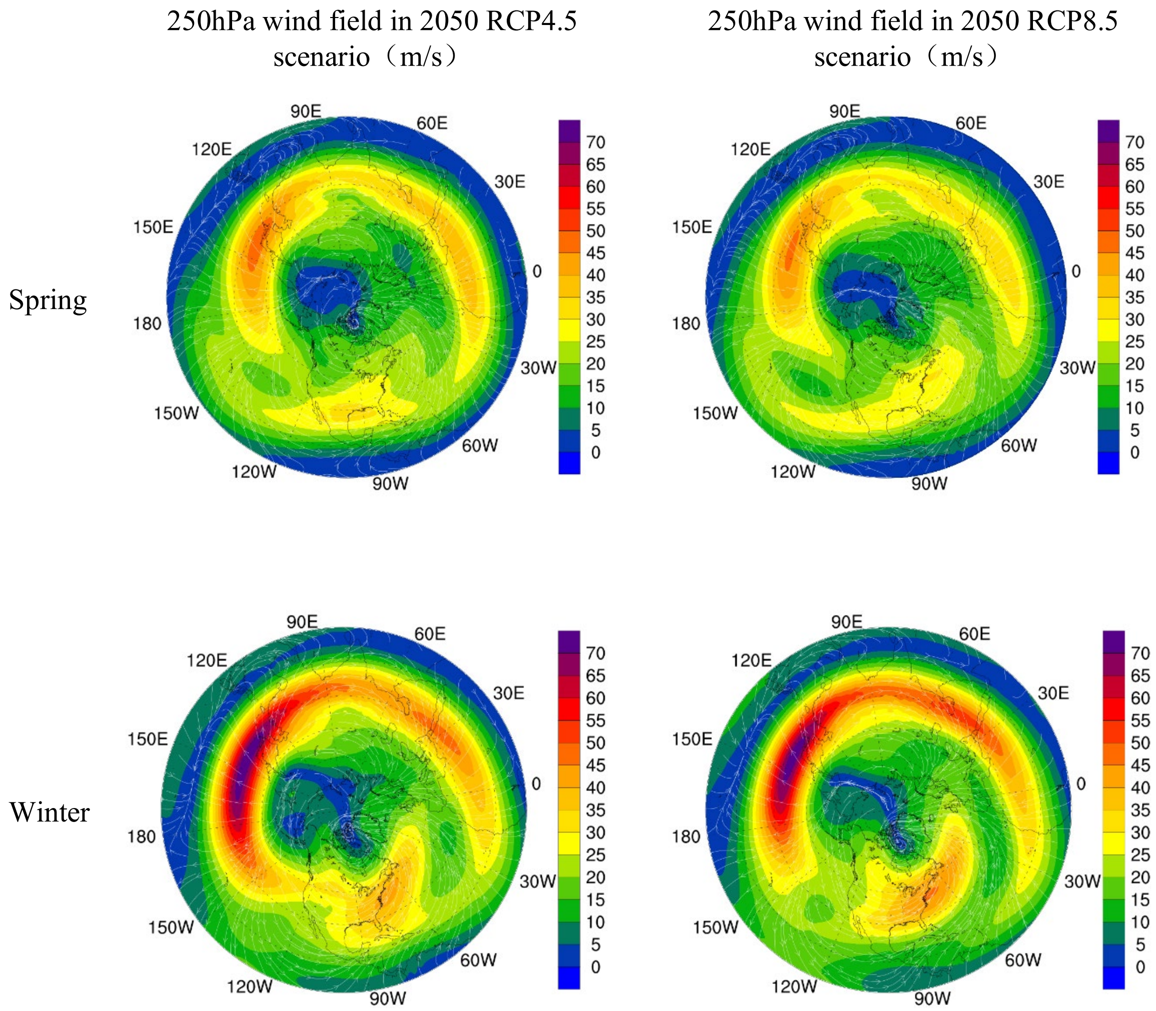


Figure S8. Wind field in 2050 RCP8.5 scenario as compared to in RCP4.5 scenario

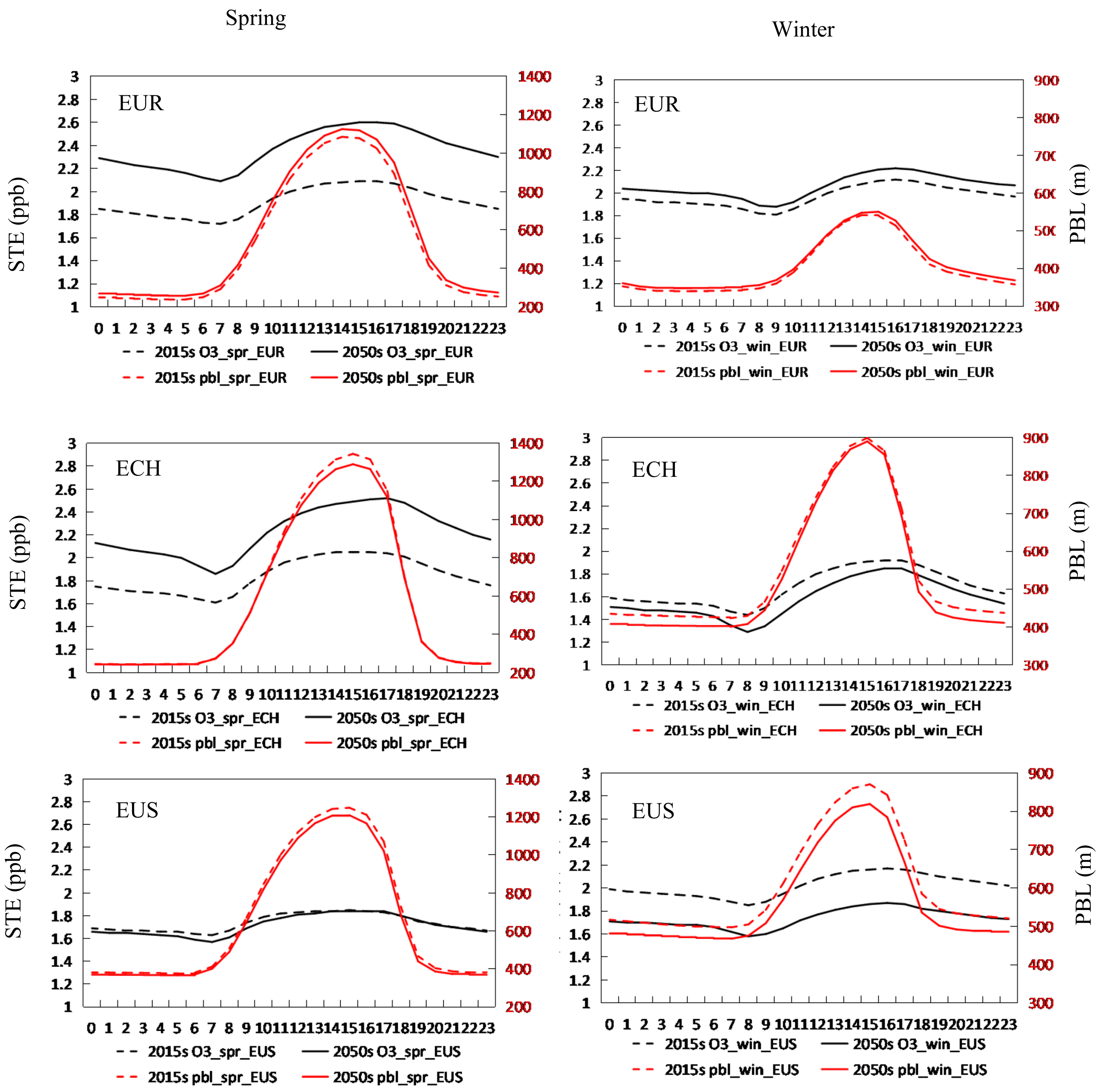


Figure S9. Hourly STE and PBL variation in winter and spring across EUR, ECH and EUS
